# Picroside II protects rat kidney against ischemia/reperfusion-induced oxidative stress and inflammation by the TLR4/NF-κB pathway

**DOI:** 10.3892/etm.2015.2225

**Published:** 2015-01-28

**Authors:** LEI WANG, XIU-HENG LIU, HUI CHEN, ZHI-YUAN CHEN, XIAO-DONG WENG, TAO QIU, LIN LIU

**Affiliations:** Department of Urology, Renmin Hospital of Wuhan University, Wuhan, Hubei 430060, P.R. China

**Keywords:** picroside II, ischemia and reperfusion injury, inflammation, oxidative stress

## Abstract

Picroside II possesses a wide range of pharmacological effects and has been demonstrated to ameliorate cerebral ischemia and reperfusion (I/R) injury. However, its effects on renal I/R injury remain unclear. In the present study, the role of picroside II in attenuating oxidative stress and the inflammatory response in a rat model of renal I/R injury was investigated. Sprague Dawley rats were subjected to 45 min of ischemia followed by 24 h of reperfusion. Prior to reperfusion, the rats were treated with picroside II or an equal volume of phosphate-buffered saline. Renal function and histological changes were compared and the relevant parameters of oxidative stress and inflammation were detected. The expression of toll-like receptor 4 (TLR4) and nuclear factor κB (NF-κB; p65) were assessed by immunohistochemistry and western blotting. It was observed that renal function was significantly improved by treatment with picroside II. Morphological analysis indicated that picroside II clearly reduced tissue damage and the expression of TLR4 and NF-κB. Reverse transcription-quantitative polymerase chain reaction demonstrated that picroside II inhibited the increase of tumor necrosis factor (TNF)-α, interleukin (IL)-1β and intercellular adhesion molecule (ICAM)-1 expression induced by I/R injury. Western blot analysis indicated that the expression levels of TLR4 and NF-κB were significantly downregulated in the picroside II group compared with those in the I/R group. These results indicate that picroside II treatment suppressed the TLR4/NF-κB signaling pathway, protecting renal tissue against I/R-induced oxidative stress and inflammatory response.

## Introduction

Renal ischemia and reperfusion (I/R) injury can increase the rates of acute kidney failure, delayed graft function and early mortality in patients undergoing kidney transplantation (1). Picroside II is one of the main active constituents isolated from *Picrorhiza scrophulariiflora*. The roots of this plant are of benefit and often used in traditional Chinese medicine for a number of conditions (2). Picroside II has been shown to possess a wide range of pharmacological effects, including effects against oxidative stress and inflammation (3–5). Picroside II has also been shown to protect against I/R in other organs, including the brain (6), due to its anti-oxidative, anti-inflammatory and anti-apoptotic properties. However, its impact on renal I/R injury remains unknown.

Inflammation is recognized as an important component of renal I/R injury (7,8). Leukocytes are the key mediators that may fuel inflammatory reactions in renal I/R injury. In the early phase of reperfusion, the levels of various pro-inflammatory cytokines increase rapidly. Oxidative stress also plays an important role in renal I/R injury (9,10). The production of reactive oxygen species (ROS) in the reperfusion period is considered to be a key reason for uncontrolled oxidative stress (11), and the increased amount of ROS can also drive the inflammatory cascade (12).

Toll-like receptors (TLRs) are a family of transmembrane proteins. Their activation leads to an intracellular cascade of events, during which nuclear factor κB (NF-κB) is released from IκB, allowing NF-κB translocation from the cytoplasm to the nucleus where it mediates the overexpression of inflammatory cytokine genes leading to a pro-inflammatory response (13). The close association between the mechanisms of renal I/R and the protection provided by picroside II suggest that picroside II may have a beneficial effect in protecting against renal I/R injury. Therefore, the major purpose of the present study was to determine whether picroside II was able to attenuate oxidative stress and inflammation following renal I/R injury and the potential underlying mechanism.

## Materials and methods

### Animal model of I/R

All surgical and experimental procedures were approved by the Institutional Animal Care and Use Committee of Wuhan University (Wuhan, China). Adult male Sprague Dawley rats (220–250 g) were obtained from the Center of Experimental Animals in Wuhan University Medical College. The procedures were carried out according to routine animal-care guidelines, and all experimental procedures complied with the Guide for the Care and Use of Laboratory Animals (1996). Briefly, rats were anesthetized with pentobarbital (45 mg/kg) and placed on a homeothermic table in order to maintain a core body temperature of 37°C. A midline laparotomy was conducted and right nephrectomy was performed. Subsequently, the left kidney was subjected to 45 min of ischemia followed by 24 h of reperfusion.

The animals were divided into three groups, namely the sham, I/R and picroside II groups. Each group contained eight rats. In the sham group, only the right kidneys were removed. In the I/R and picroside II groups, the left kidney vessels were clamped for 45 min followed by 24 h of reperfusion. The interventions were performed as described below.

### Intervention study

Picroside II (CAS No: 39012-20-9, purity >98%, molecular formula C_23_H_28_O_13_) was purchased from Tianjin Kuiqing Medical Technology Co., Ltd. (Tianjin, China). It was diluted to form a 10 g/l solution with 1 mol/l phosphate-buffered saline (PBS). Picroside II (10 mg/kg) 250 μl was administered via the tail vein to rats in the picroside II group with a micro-syringe according to a previous study (6), at the end of the 45 min of ischemia and prior to 24 h of reperfusion. The rats in the I/R and sham groups were simultaneously injected with 250 μl 1 mol/l PBS. Following the 24-h reperfusion period, the animals were sacrificed with an overdose of pentobarbital sodium (Sigma-Aldrich, St. Louis, MO, USA), and the left kidneys were removed for the following experiments and blood samples were collected for the detection of blood urea nitrogen (BUN) and creatinine (Cr) levels.

### Preservation of kidneys

The left kidney was removed under fully maintained anesthesia. After removal, the kidney was fixed in 10% phosphate-buffered formalin or immediately frozen, and stored at −80°C for subsequent experiments.

### Serum assays

At 24 h after I/R injury in every group, 1 ml blood samples were taken and analyzed according to the instructions of Creatinine and Urea Assay kits (Nanjing Jiancheng Bioengineering Institute, Nanjing, China). The absorbance was measured using a spectrophotometer (UV-1700; Shimadzu Corporation, Tokyo, Japan) and then the concentrations of BUN and Cr were calculated.

### Histologic examination

After the kidney fixed in 10% phosphate-buffered formalin, it was embedded with paraffin and sectioned at 4 μm thickness. The sections were deparaffinized and hydrated gradually, and then stained with hematoxylin and eosin (H&E). Morphologic assessments were conducted by an experienced renal pathologist who was unaware of the treatments. An established grading scale of 0–4, outlined by Jablonski *et al* (14), was used for the histopathological assessment of I/R-induced damage.

### Assay of malondialdehyde (MDA) and superoxide dismutase (SOD)

The frozen samples of the ischemic zone were homogenized and centrifuged at 3,000 × g for 10 min. Subsequently, the supernatants were collected for analysis of the MDA level and SOD activity. The measurements were obtained spectrophotometrically with commercial SOD and MDA Assay kits (Nanjing Jiancheng Bioengineering Institute) according to the manufacturer’s instructions. The MDA level was represented in nmol/mg protein. The SOD activity was expressed as U/mg protein.

### Immunohistochemistry

The expression of TLR4 and NF-κB was examined by immunohistochemical staining. Briefly, 4-μm sections were deparaffinized, and endogenous peroxidase activity was blocked with 3% hydrogen peroxide at 37°C for 10 min. The sections were then treated with 10% normal goat serum in Tris-buffered saline for 30 min at 37°C. Subsequently, they were incubated overnight at 4°C with monoclonal anti-rat anti-TLR4 (1:100; ab8376; Abcam, Cambridge, MA, USA) and monclonal anti-rat anti-NF-κB (1:100; sc-8008, Santa Cruz Biotechnology, Santa Cruz, CA) antibodies. After washing three times with PBS, these sections were incubated with the secondary antibody from the UltraVision™ Quanto Detection System HRP DAB (Thermo Fisher Scientific, Waltham, MA, USA) for 30 min at room temperature, followed by the color reagent 3,3′-diaminobenzidine (DAB). In the negative control group, the experiments were routinely performed.

### Reverse transcription-quantitative polymerase chain reaction (RT-qPCR)

Total RNA was isolated using TRIzol reagent (Invitrogen Life Technologies, Carlsbad, CA, USA) and the RNA concentration was obtained by spectrophotometry. Single-stranded cDNA was synthesized using the cDNA synthesis kit (Takara, Kyoto, Japan) according to the manufacturer’s instructions. qPCR was performed with the Applied Biosystems SYBR Green mix kit (Applied Biosystems, CA, USA). The PCR reaction mixture contained: 2 μl cDNA, 12.5 μl 2X SYBR Green mix, 1μl forward primer, 1μl reverse primer and 8.5 μl ddH_2_O, in a final volume of 25 μl. The primers used were as follows: Tumor necrosis factor (TNF)-α forward, 5′-CTTCTCATTCCTGCTCGTGG-3′ and reverse, 5′-TCCGCTTGGTGGTTTGCTAC-3′ (Gen-Bank accession number NM_012675.3); interleukin (IL)-1β forward, 5′-ACTATGGCAACTGTCCCTGAAC-3′ and reverse, 5′-GTGCTTGGGTCCTCATCCTG-3′ (Gen-Bank accession number NM_031512.2); intercellular adhesion molecule (ICAM)-1 forward, 5′-GGGATGGTGAAGTCTGTCAA-3′, and reverse, 5′-GGCGGTAATAGGTGTAAATGG-3′ (Gen-Bank accession number NM_012967). β-actin was used as a reference gene. The data are presented as a ratio of the mRNA of the gene of interest to β-actin mRNA (sense: 5′-TGCTATGTTGCCCTAGACTTCG-3′ and antisense: 5′-GTTGGCATAGAGGTCTTTACGG-3′ and NM_031144).

### Western blot analysis

Total proteins were extracted, and quantified using the bicinchoninic acid method. Then, equivalent weights of protein (40 μg/lane) were separated on 10% SDS-PAGE gels and transferred to a nitrocellulose membrane. The membranes were blocked with 5% non-fat milk in Tris-buffered saline and Tween 20 buffer and then incubated with the following primary antibodies: mouse monclonal NF-κB (p65; 1:500 dilution; sc-8008, Santa Cruz Biotechnology) and rabbit polyclonal TLR4 (1:500 dilution; sc-10741, Santa Cruz Biotechnology) at 4°C overnight. Subsequently, after being washed twice with PBS, the membranes were incubated with horseradish peroxidase (HRP)-conjugated goat anti-rabbit (ZDR-5306) and goat anti-mouse (ZDR-5307) secondary antibodies (1:2,000; ZSGB-BIO, Beijing, China). Specific bands were visualized using an enhanced chemiluminescence detection kit (Immobilon Western Chemiluminescence HRP Substrate; Merck Millipore, Darmstadt, Germany). Optical densities were detected using Quantity One software (Bio-Rad Laboratories, Hercules, CA, USA).

### Statistical analysis

Data are presented as mean ± standard error of the mean. The means of the different groups were compared using one-way analysis of variance and Student-Newman-Keuls test. Statistical analyses were conducted using SPSS version 17.0 (SPSS Inc., Chicago, IL, USA). Differences were considered statistically significant when P<0.05.

## Results

### Renal function

**Figure 1:**
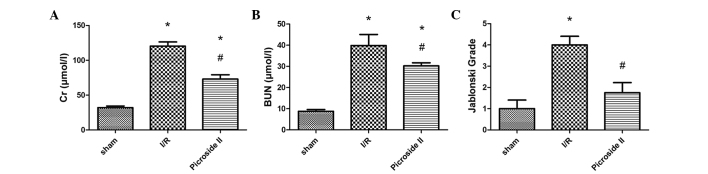
Effect of picroside II on (A) serum Cr concentration, (B) serum BUN concentration and (C) Jablonski grading scale scores after 45 min of ischemia followed by 24 h of reperfusion. Bars represent means ± standard error of the mean (n=4 per group); ^*^P<0.05 vs. the sham group, ^#^P<0.05 vs. the I/R group. Cr, creatinine, BUN, blood urea nitrogen; I/R, ischemia and reperfusion.

It was evident from the results of the serum assays that the rats subjected to I/R injury exhibited significant increases in BUN and Cr levels compared with rats in the sham group. The renal function changes induced by I/R were significantly ameliorated by treatment with picroside II (Fig. 1A and B).

### Histopathology

**Figure 2:**
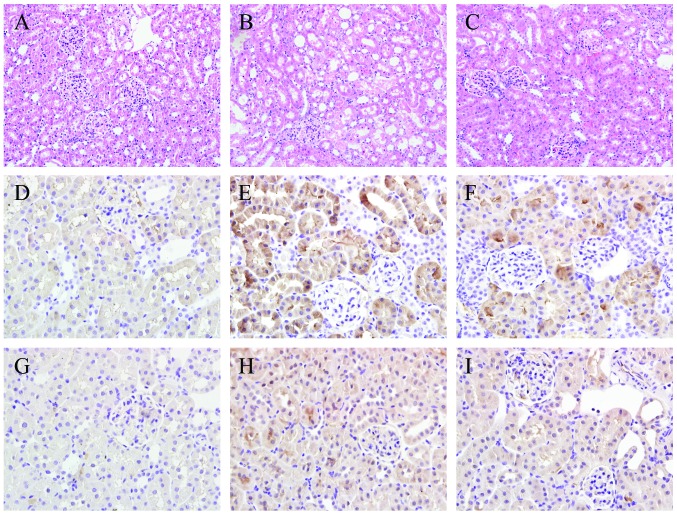
Histologic features were evaluated by H&E and immunohistochemical staining. (A–C) Representative kidney sections stained with H&E. (D–F) TLR4 expression in the kidneys following 24 h of reperfusion. (G–I) NF-κB expression in the kidneys following 24 h of reperfusion. (A, D, G) Sham group: Sections from a sham-operated rat. (B, E, H) I/R group: Section from rat subjected to I/R treatment. (C, F, I) Picroside II group: Section from rat subjected to I/R and treated with picroside II. H&E staining, original magnification ×200; Immunohistochemical staining, original magnification ×400. H&E, hematoxylin and eosin. TLR, toll-like receptor; NF-κB, nuclear factor-κB; I/R, ischemia and reperfusion.

Renal I/R resulted in significant renal injury, as evidenced by tubular necrosis, medullary hemorrhage and congestion. However, treatment with picroside II reduced the severity of the renal damage (Fig. 2A–C). According to Jablonski scores, 45 min of renal ischemia followed by 24 h of reperfusion resulted in severe acute tubular necrosis. Quantitative analysis showed a significantly decreased score in the picroside II group compared with the I/R group (Fig. 1C).

### MDA and SOD analysis

**Figure 3:**
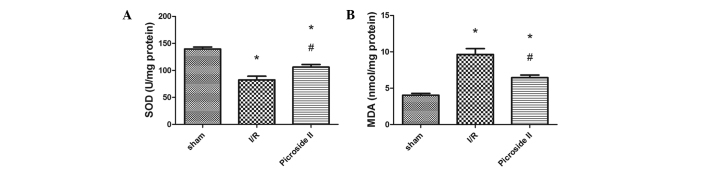
Effect of picroside II on (A) SOD activity and (B) MDA content after 45 min of ischemia followed by 24 h of reperfusion. Bars represent means ± standard error of the mean (n=4 per group); ^*^P<0.05 vs. the sham group, ^#^P<0.05 vs. the I/R group. SOD, superoxide dismutase; MDA, malondialdehyde; I/R, ischemia and reperfusion.

As shown in Fig. 3A, the SOD activity decreased markedly in the I/R group compared with that in the sham group; however, picroside II inhibited the reduction of SOD activity that was induced by I/R injury. The MDA content in the I/R group was significantly higher than that in the sham group, whereas this increase induced by I/R injury was significantly attenuated following treatment with picroside II.

### Immunohistochemistry

The expression of TLR4 (Fig. 2D–F) and NF-κB (Fig. 2G–I) was detected by immunohistochemical staining. The results revealed that TLR4 and NF-κB positive cells were rarely found in the sham group. However, in the I/R group, renal tissues were strongly positive for TLR4 and NF-κB expression. These expression spots were reduced in the picroside II group compared with those in the I/R group.

### RT-qPCR analysis

**Figure 4:**
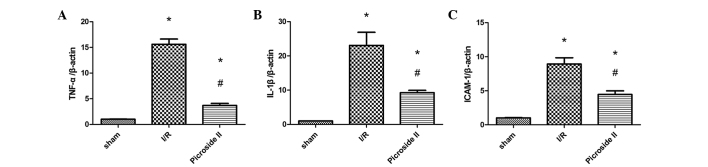
mRNA levels of TNF-α, IL-1β and ICAM-1 in the kidney. Effects of picroside II on the mRNA level of (A) TNF-α, (B) IL-1β and (C) ICAM-1 after 45 min of ischemia followed by 24 h of reperfusion. mRNA was standardized for β-actin mRNA. Bars represent means ± standard error of the mean (n=4 per group); ^*^P<0.05 vs. the sham group, ^#^P<0.05 vs. the I/R group. TNF, tumor necrosis factor; IL, interleukin; ICAM, intercellular adhesion molecule; I/R ischemia and reperfusion.

The relative mRNA expression levels of TNF-α, IL-1β and ICAM-1 to β-actin were evaluated. The mRNA levels of TNF-α, IL-1β and ICAM-1 were significantly greater in the I/R group than in the sham group. However, treatment with picroside II significantly reduced the mRNA expression levels of TNF-α, IL-1β and ICAM-1 following I/R (Fig. 4).

### Western blot analysis

**Figure 5:**
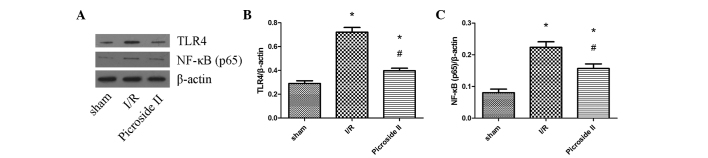
Representative western blots showed the effects of picroside II on TLR4 and NF-κB (p65) expression in the kidney after 45 min of ischemia followed by 24 h of reperfusion. β-actin was used to show equal amounts of protein loading in each lane. (A) Representative western blots showing the effects of picroside II on TLR4 and NF-κB expression. Relative band densities of (B) TLR4 and (C) NF-κB to the mean value of the control. Bars represent means ± standard error of the mean (n=4 per group); ^*^P<0.05 vs. the sham group, ^#^P<0.05 vs. the I/R group.

The levels of TLR4 and NF-κB (p65) protein expression were measured by western blotting (Fig. 5). It was clear from the results that the expression levels of TLR4 and NF-κB were upregulated in the I/R and picroside II groups when compared with those in the sham group. However, picroside II attenuated these I/R-induced increases in expression.

## Discussion

In this study, it was demonstrated that the protective effects of picroside II in the kidney were associated with a reduction of oxidative stress and inflammation. Picroside II is one of the main active constituents of the extracts of *Picrorhiza scrophulariiflora* Pennell and has been shown to possess a wide range of pharmacological effects, including neuroprotective, hepatoprotective, anti-apoptosis, anti-cholestatic, anti-inflammatory and immune-modulating activities (3–5). In a previous study, it was demonstrated that picroside II was able to inhibit apoptosis in rats subjected to middle cerebral I/R injury (6). Another study reported that picroside II protected hepatocytes against injury through maintaining the integrity of the mitochondrial membrane and enhancing the activity of ATPase in mitochondria (15). However, it has not previously been demonstrated whether picroside II is able to protect tissue against renal I/R injury. In the present study, it was demonstrated for the first time to the best of our knowledge, that picroside II reduced the oxidative stress and inflammation induced by renal I/R injury in rats.

Renal injury caused by I/R has a complicated pathological course. Inflammation represents a key factor in the occurrence and development of ischemic damage, which is considered to occur secondary to an intense inflammatory response initiated by the infiltration of leukocytes and the production of pro-inflammatory cytokines following I/R (16). Within hours after ischemia, the infiltrating leukocytes may release a large number of pro-inflammatory mediators that contribute to the development of tissue damage. TNF-α and IL-1β are key inflammatory cytokines participating in the pathological process of I/R injury. Due to the assistance of TNF-α, the infiltration of leukocytes into the kidney may aggravate ischemic injury (17). IL-1β is an immune-derived cytokine and can promote the secretion of itself under ischemic stimuli, the so-called autocrine-like function (17). ICAM, as an adhesion molecule, can facilitate leukocyte infiltration and adhesion to aggravate the injuries caused by I/R. In the present study, it was observed that the mRNA levels of TNF-α, IL-1β and ICAM-1 were significantly greater in the I/R group than in the sham group. However, these increased markers of inflammation were reduced by treatment with picroside II (Fig. 4).

ROS play a key role in the development of renal I/R injury. They can cause direct damage to membranes and proteins as well as indirect damage through the activation of pro-apoptotic pathways (18). Normally, the generated ROS from metabolic processes can be scavenged by endogenous antioxidant enzymes such as SOD (19), catalyzing the dismutation of the superoxide radical to hydrogen peroxide. MDA, as a significant product of lipid oxidation (20), is often used to reflect the extent of cell injury by oxidative stress. It has been reported that reducing the elevation of MDA levels and suppressing the reduction of SOD activity has protective effects in cardiac I/R injury (21). The present study revealed that picroside II administration significantly reduced MDA level elevation and attenuated the reduction of SOD activity in renal I/R injury.

Through MyD88, TLR4 signals can lead to the subsequent downstream activation of NF-κB (p65) and mitogen-activated protein kinase signaling pathways (22). NF-κB, an important nuclear transcription factor, regulates the expression of a large number of genes, which play key roles in the regulation of apoptosis, inflammation, viral replication and tumorigenesis (23). Numerous stimuli, including I/R injury, can activate NF-κB signaling by the degradation of IκB and release of the NF-κB p65-p50 dimer, which translocates to the nucleus, binds to κB binding sites on DNA, and regulates the transcriptional activation of target genes (24). The current study demonstrated that renal tubular injury and inflammatory cell infiltration were markedly alleviated in rats following picroside II treatment. The expression levels of TLR4 and NF-κB significantly decreased in the picroside II group compared with those in the I/R group, as did BUN and Cr levels. This suggests that picroside II may alleviate I/R-induced oxidative stress and inflammatory response by blocking the TLR4/NF-κB pathway.

In conclusion, this study demonstrated for the first time that the administration of picroside II had a protective effect on renal I/R injury, which may be ascribed to blockade of the TLR4/NF-κB pathway resulting in attenuation of oxidative stress and the inflammatory response.

## Abbreviations

MDA: malondialdehyde
SOD: superoxide dismutase
I/R: ischemia and reperfusion
